# Effectiveness of teriflunomide on No Evidence of Disease Activity and cognition in relapsing remitting multiple sclerosis: results of the NEDA3PLUS study

**DOI:** 10.1007/s00415-023-11820-0

**Published:** 2023-07-05

**Authors:** Maria Pia Amato, Roberto Bergamaschi, Diego Centonze, Massimiliano Mirabella, Girolama Alessandra Marfia, Rocco Totaro, Giacomo Lus, Vincenzo Brescia Morra, Umberto Aguglia, Cristoforo Comi, Paola Cavalla, Mauro Zaffaroni, Marco Rovaris, Luigi Maria Grimaldi, Stefania Leoni, Simona Malucchi, Eleonora Baldi, Marcello Romano, Mario Falcini, Paola Perini, Maurizio Assetta, Emilio Portaccio, Sergio Sommacal, Nunzio Olivieri, Franco Parodi, Daniele Santo Todaro, Nicoletta Grassivaro, Alberto Farina, Margaret Mary Mondino, Massimo Filippi, Maria Trojano

**Affiliations:** 1 Department of NEUROFARBA, Section of Neurosciences, University of Florence, Florence, Italy; 2 IRCCS Fondazione Don Carlo Gnocchi, Florence, Italy; 3 IRCCS Mondino Foundation, Pavia, Italy; 4 Unit of Neurology and Neurorehabilitation, IRCCS Neuromed, Pozzilli, Italy; 5 Fondazione Policlinico Universitario ‘Agostino Gemelli’ IRCCS, Neurology Unit, Rome, Italy; 6 Centro di Ricerca Sclerosi Multipla (CERSM), Università Cattolica del Sacro Cuore, Rome, Italy; 7 Multiple Sclerosis Clinical and Research Unit, Department of Systems Medicine, University of Rome Tor Vergata, Rome, Italy; 8 Demyelinating Disease Center, San Salvatore Hospital, L’Aquila, Italy; 9 Department of Advanced Medical and Surgical Sciences, University of Campania Luigi Vanvitelli, Naples, Italy; 10 Department of Neuroscience, Reproductive Science and Odontostomatology, University Federico II, Multiple Sclerosis Clinical Care and Research Centre, Naples, Italy; 11 Department of Medical and Surgical Sciences, “Magna Graecia” University, Catanzaro, Italy; 12 Regional Epilepsy Centre, Great Metropolitan Hospital, Reggio Calabria, Italy; 13 Department of Translational Medicine and Interdisciplinary Research Center of Autoimmune Diseases, University of Piemonte Orientale, Novara, Italy; 14 Department of Neuroscience and Mental Health, City of Health and Science University Hospital of Turin, Multiple Sclerosis Center, Turin, Italy; 15 ASST della Valle Olona, Hospital of Gallarate, Neuroimmunology Unit, Gallarate, Italy; 16 IRCCS Fondazione Don Carlo Gnocchi Onlus, Multiple Sclerosis Center, Milan, Italy; 17 Foundation Institute “G. Giglio”, MS Center, Cefalù-Palermo, Italy; 18 Unit of Clinical Neurology, AOU Sassari, Sassari, Italy; 19 University Hospital San Luigi Gonzaga, SCDO Neurologia-CRESM, Orbassano, Turin, Italy; 20 Department of Neuroscience and Rehabilitation, S. Anna Hospital, Multiple Sclerosis Center, Ferrara, Italy; 21 Neurology and Stroke Unit, Villa Sofia Cervello Hospital, Palermo, Italy; 22 Santo Stefano Hospital, Neurology Unit, Prato, Italy; 23 University Hospital of Padua, Multiple Sclerosis Centre of the Veneto Region (CeSMuV), Padua, Italy; 24 Department of Neurology, ‘G. Mazzini’ Hospital, Teramo, Italy; 25 Medical Department, Sanofi, Milan, Italy; 26 IRCCS San Raffaele Scientific Institute, Neurology Unit, Milan, Italy; 27 IRCCS San Raffaele Scientific Institute, Neurorehabilitation Unit, Milan, Italy; 28 IRCCS San Raffaele Scientific Institute, Neurophysiology Service, Milan, Italy; 29 Division of Neuroscience, IRCCS San Raffaele Scientific Institute, Neuroimaging Research Unit, Milan, Italy; 30 Vita-Salute San Raffaele University, Milan, Italy; 31 School of Medicine, University “Aldo Moro” of Bari, Bari, Italy

**Keywords:** Multiple sclerosis, Teriflunomide, No Evidence of Disease Activity, Cognition, Observational

## Abstract

**Background:**

Cognitive impairment (CI) is a prevalent and debilitating manifestation of multiple sclerosis (MS); however, it is not included in the widely used concept of No Evidence of Disease Activity (NEDA-3). We expanded the NEDA-3 concept to NEDA-3 + by encompassing CI assessed through the Symbol Digit Modality Test (SDMT) and evaluated the effect of teriflunomide on NEDA3 + in patients treated in a real-world setting. The value of NEDA-3 + in predicting disability progression was also assessed.

**Methods:**

This 96-weeks observational study enrolled patients already on treatment with teriflunomide for ≥ 24 weeks. The predictiveness of NEDA-3 and NEDA-3 + at 48 weeks on the change in motor disability at 96 weeks was compared through a two-sided McNemar test.

**Results:**

The full analysis set (n = 128; 38% treatment naïve) featured relatively low level of disability (baseline EDSS = 1.97 ± 1.33). NEDA-3 and NEDA-3 + statuses were achieved by 82.8% and 64.8% of patients, respectively at 48 weeks vs. baseline, and by 57.0% and 49.2% of patients, respectively at 96 weeks vs. baseline. All patients except one were free of disability progression at Week 96, and NEDA-3 and NEDA-3 + were equally predictive. Most patients were free of relapse (87.5%), disability progression (94.5%) and new MRI activity (67.2%) comparing 96 weeks with baseline. SDMT scores were stable in patients with baseline score ˃35 and improved significantly in those with baseline score ≤ 35. Treatment persistence was high (81.0% at Week 96).

**Conclusion:**

Teriflunomide confirmed its real-world efficacy and was found to have a potentially beneficial effect on cognition.

**Supplementary Information:**

The online version contains supplementary material available at 10.1007/s00415-023-11820-0.

## Introduction

Multiple sclerosis (MS) is an immune-mediated, chronic, demyelinating disease of the central nervous system that affects approximately 2.8 million people worldwide [1]. Although the course of the disease is largely unpredictable, approximately 85% of people with MS begin with episodes of reversible neurological deficits (relapsing–remitting MS), which are often followed by progressive neurological deterioration over time [2].

Several disease-modifying therapies (DMTs) have been approved to successfully reduce the occurrence of relapses and slow progression. Teriflunomide is a once-daily, oral immunomodulatory agent that selectively and reversibly inhibits the mitochondrial enzyme dihydroorotate dehydrogenase, blocking de novo pyrimidine synthesis and reducing B and T lymphocyte proliferation [3, 4]. The efficacy and safety of teriflunomide were demonstrated in both pivotal phase 3 randomized placebo-controlled trials [5–7] and their long-term extension studies [8, 9]. When our study was designed (2017), little real-world evidence was however available supporting the use of teriflunomide, although the Teri-PRO study did demonstrate high treatment satisfaction, along with stability in disability, cognition, quality of life outcomes, and a manageable safety and tolerability profile [10].

No Evidence of Disease Activity (NEDA-3), defined as an absence of relapses, disability progression lasting at least 3 months and no new MRI lesions has become a new goal and outcome measure for MS treatment. A recent systematic review and meta-analysis of studies conducted between 1 January 2006 and 26 January 2021 found that NEDA-3 is associated with no long-term disability progression in RRMS patients on both low- and high-efficacy therapy [11]. Several post-hoc exploratory analyses have investigated the efficacy of oral DMTs versus placebo in achieving NEDA-3 status [12–17]. In a post hoc analysis of the teriflunomide TEMSO study, a greater proportion of patients treated with teriflunomide 7 or 14 mg were free from disease activity than individuals receiving placebo [18].

Cognitive impairment (CI) is present in a large proportion (40–70%) of people with MS and has a negative impact on performance in everyday activities including employment, social interactions, treatment adherence, and functional independence. MS typically affects information processing speed, memory (episodic, working and semantic), and executive function domains. CI is important also from a therapeutic perspective since cognitively impaired patients are less able to understand explanations about the disease and treatments, are less adherent to therapy, and may be less reliable in reporting their symptoms [19].

Cognition should be assessed in all MS patients during everyday clinical evaluation using currently available and validated instruments. Among these, the Symbol Digit Modality Test (SDMT) is particularly suitable as it is fast (5 min), easily reproducible, and does not require specific neuropsychological training for its administration. Compared to the other tests, it is more sensitive, requires less time, does not require any electronic equipment, and has a prognostic value correlating with the degree of disability at 5 and 7 years [20].

Given the importance of CI in MS, NEDA-3 may provide an incomplete picture of disease activity. Collectively, relapses, MRI-lesion activity, and worsening of disability provide useful information about inflammatory activity in the brain but may not adequately account for disease progression [21]. Although neurodegenerative damage may be captured in part by assessing disability worsening based on changes in Expanded Disability Status Scale (EDSS) score, other aspects of disease progression such as cognitive decline and fatigue may be overlooked. One proposal for bridging this gap is to expand the NEDA-3 concept to a four-domain evaluation, NEDA-3 + , which assesses CI through the SDMT in addition to relapses, disability worsening, and MRI-lesion activity.

At the time of this study, there was no information on NEDA-3 and NEDA-3 + or on their use as predictors of treatment response in a “real life” population of MS patients treated with teriflunomide in day-to-day clinical practice. Moreover, there were no real-world data about the capacity of these tools to predict motor disability in the medium term. The aims of the present study were thus to evaluate the effect of teriflunomide on NEDA-3 + in RRMS patients in a real-world setting, and to assess the value of NEDA-3 + in predicting disability progression.

## Methods

**Fig. 1:**
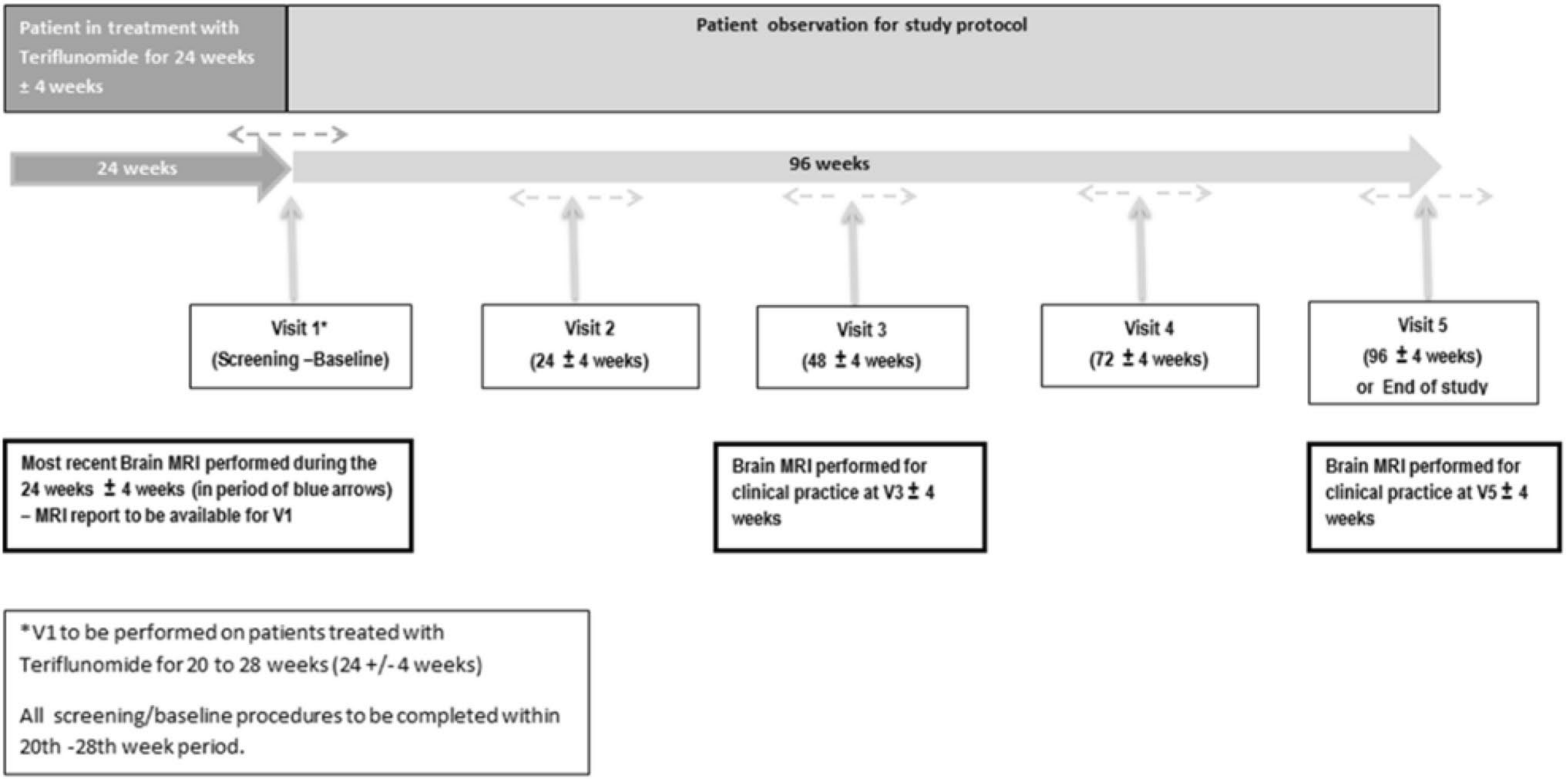
Study design

This was a prospective, non-interventional study involving patients treated with teriflunomide under real-world conditions. Visits were held at Week 0 (Screening/Baseline, Visit 1), Week 24 (Visit 2), Week 48 (Visit 3), Week 72 (Visit 4) and Week 96 (Visit 5). The study was conducted at 22 sites located in Italy and included adult consenting patients with EDSS ≤ 5.5 who had already been on treatment with teriflunomide for 24 ± 4 weeks. Figure 1 illustrates the study design.

NEDAs were calculated at 48 weeks vs baseline, 96 weeks vs 48 weeks, 96 weeks vs baseline. Secondary efficacy variables included the proportion of patients who were free of relapse, new MRI activity or disability and annualized relapse rate (ARR). Fatigue Severity Scale (FSS), Beck Depression Inventory (BDI) and treatment persistence were also assessed. Definitions of the study variables are provided below:

- NEDA-3: absence of clinical relapse, 24-week Sustained Disease Progression (SDP) and MRI activity [22].
- NEDA-3 + : absence of clinical relapse, 24-week SDP, MRI activity and deteriorating cognitive performance assessed by the SDMT [23].
- NEDA-2: absence of clinical relapse and 24-week SDP [24].
- NEDA-2 + : absence of clinical relapse, 24-week SDP and deteriorating cognitive performance (SDMT).
- SDP: an increase of EDSS ≥ 1.5 points if reference timepoint score was 0, ≥ 1.0 point if reference timepoint score was 1.0–5.5 and ≥ 0.5 points if reference timepoint score was ≥ 6.0.
- SDMT raw score: range: 0–110; a drop of at least 4 points or 10% reduction of total score is considered a significant change in SMDT [25].
- SDMT T-score calculated adjusting raw score for age, gender and years of education. Cut-off value for impaired function: 35 [25].
- Beck Depression Inventory (BDI): range: 0–39; cut-off values: no depression = 0–9, mild depression = 10–19, moderate depression = 20–29, severe depression = 29–39.
- Fatigue Severity Scale (FSS): range: 0–7; cut-off value for presence of fatigue: 4.6.

The primary endpoint, the predictiveness of NEDA-3 and NEDA-3 + (raw score) at 48 weeks on the change in motor disability at 96 weeks evaluated by EDSS, (i.e., the proportion of patients without 24-week SDP at 96 weeks vs. 48 weeks), was compared by means of a two-sided McNemar test. Additionally, results are reported as absolute risk reduction (difference in proportions) with associated 95% CI. The following null and alternative hypotheses of superiority in terms of the difference were considered: H0: |PT – PS|= 0 versus H1: |PT – PS|> 0. Where PT and PS are the proportions of correct predictions for NEDA-3 + and NEDA-3, respectively. To demonstrate superiority of NEDA-3 + , the null hypothesis that NEDA-3 + and NEDA-3 are not different in predicting the evolution of motor disability was to be rejected (p value < 0.05). Estimates of the ARR at 48 and 96 weeks vs. baseline and at 96 weeks vs. 48 weeks together with associated 95% CI were derived from an analysis of the number of relapses with the use of a Poisson regression model with visit as fixed effect, the log of time during treatment serving as an offset variable and with generalized estimating equations parameterized with an unstructured correlation matrix to consider correlation between repeated measures. Analyses were performed using the GENMOD Procedure of SAS version 9.4. Time to MS-related disability (i.e., EDSS worsening) was estimated as survivor function using the Kaplan–Meier approach. Analyses were performed using the LIFETEST Procedure of SAS version 9.4. Changes from baseline of cognitive impairment, depressive symptoms and fatigue scores at 48 and 96 weeks were fitted in a mixed linear model with visit as fixed effect and a variance–covariance matrix of unstructured form to consider correlation between repeated measures. Results are reported as least-square means with associated 95% CI. Analyses were performed using the MIXED Procedure of SAS version 9.4. Shift tables were used to compare the proportions of patients fatigue-free and depression-free obtained at baseline with those obtained at each visit. Other secondary endpoints are described by means of descriptive statistics.

Analyses were performed on the enrolled population (ENR) (all consented patients), the Full Analysis Set (FAS) (all consented patients who received at least one dose of teriflunomide and had all primary endpoint assessments, regardless of compliance with the study protocol) and the safety population (SAF) (all consented patients who received at least one dose of teriflunomide).

## Results

### Participants

A total of 210 patients provided written informed consent and were enrolled. Forty-five patients (21.4%) discontinued treatment due to lack of efficacy (MRI) (31.1%), loss to follow up (24.4%), adverse event (13.3%), lack of efficacy (relapse) (13.3%), patient withdrawal (4.4%), medical decision (2.2%), lack of efficacy (disability) (2.2%), lack of efficacy (other reasons) (4.4%), pregnancy (2.2%), and other reasons (2.2%). 16.3% of naïve patients and 24.6% of non-naïve patients discontinued treatment, reasons for discontinuation are reported in the Supplementary Table 1. The study was completed by 160 patients (76.2%) and 128 (61.0%) were included in the FAS. Fifty patients (23.8% of ENR) discontinued the study; of these, 21 patients (42.0%) discontinued due to teriflunomide lack of efficacy, 7 (14.0%) for adverse events and 22 (44.0%) for other reasons. Seven patients discontinued the study but did not discontinue treatment and two patients completed the study and discontinued treatment on the last study day.

Baseline characteristics of the enrolled population are shown in Table 1. The following statistically significant differences (p ˂ 0.001) were observed stratifying for naïve/non-naïve status: previously treated patients were younger at diagnosis (34.8 ± 8.8 vs 45.0 ± 9.9 years) had a longer mean time from onset of MS symptoms (14.9 ± 9.2 vs 6.7 ± 8.7 years), time from MS diagnosis (11.8 ± 8.0 vs 2.9 ± 5.8 years) and time from onset of last relapse (median 5.1 vs 0.8 years). Baseline EDSS was also greater in non-naïve patients (2.10 ± 1.38 vs 1.76 ± 1.23 [p = 0.07]). Most previously treated patients had no relapses in the previous or last two years, whereas the majority of naïve patients had one relapse. The most common reasons for switching to teriflunomide in previously treated patients were poor tolerability (51%) and lack of efficacy (15%). Most patients switched from glatiramer acetate, interferon beta or dimethyl fumarate. No noteworthy differences were seen stratifying by gender or SDMT score at baseline (data not shown). Baseline characteristics of the FAS were similar to those of the enrolled population and are available in Supplementary Table 2.

**Table 1:** Baseline disease characteristics (ENR population)

Baseline	All (n = 210)	Naïves (n = 80)	Non-naïves (n = 130)
Age, mean years ± SD [range]	47.0 ± 8.6 [21; 69]	47.8 ± 9.1 [21; 67]	46.5 ± 8.2 [22; 69]
Female (%)	71.4	65.0	75.3
Caucasian (%)	100	100	100
DMT naïve (%)	38.0	100	0
Education, mean years ± SD [range]	12.5 ± 3.6 [5;20]	12.3 ± 3.3 [8; 20]	12.6 ± 3.6 [5; 18]
Time from			
• Symptoms onset, mean years (SD)	11.8 (9.9)	6.7 (8.7)	14.9 (9.2)*
• Diagnosis, mean years (SD)	8.4 (8.4)	2.9 (5.8)	11.8 (8.0)*
• Onset of last relapse, median years (IQR)	1.6 (0.8–6.8)	0.8 (0.6–1.4)	5.1 (1.4–9.0)*
EDSS, mean (SD)	1.97 (1.33)	1.76 (1.23)	2.10 (1.37)
No. of relapses in previous year (% of enrolled population with available data)	n = 204	n = 124	n = 80
• 0	60.8	37.5	75.8 *
• 1	35.3	56.2	21.8 *
• ≥ 2	3.9	6.2	2.4 *
No. of relapses in the last 2 years (% of enrolled population with available data)	n = 203	n = 124	n = 79
• 0	48.3	17.7	67.7*
• 1	40.9	65.8	25.0*
• ≥ 2	10.8	16.5	7.3*
Severity of last relapse (%)			
• Mild	63.3	62.5	63.8
• Moderate	34.8	36.2	33.8
• Severe	1.9	1.2	2.3
SDMT T-score, mean (SD)	45.1 (13.4)	46.63 (13.67)	44.04 (13.21)
BDI, mean (SD)	6.5 (6.2)	5.89 (5.42)	6.79 (6.54)
FSS, mean (SD)	3.9 (1.8)	3.68 (1.83)	4.01 (1.71)

*p < 0.0001 vs. Naïves

### Relapses

In the FAS, 94.5% of patients (95% CI 89.1; 97.8) were free of relapse at Week 48 vs. baseline, 87.5% (95% CI 80.5; 92.7) at Week 96 vs. baseline, and 90.6% (95% CI 84.2; 95.1) at Week 96 vs. Week 48. A significantly greater proportion of naïve than previously treated patients were relapse free when comparing Week 96 to baseline (96.1% vs 81.8%; p = 0.0169) and Week 96 to Week 48 (98.0% vs. 85.7%; p = 0.0192). No significant differences were observed stratifying by gender and SDMT score. The ARR was 0.039 (95% CI 0.019; 0.081) at 48 weeks and 0.029 (95% CI 0.015; 0.056) at 96 weeks.

### Disability

**Fig. 2:**
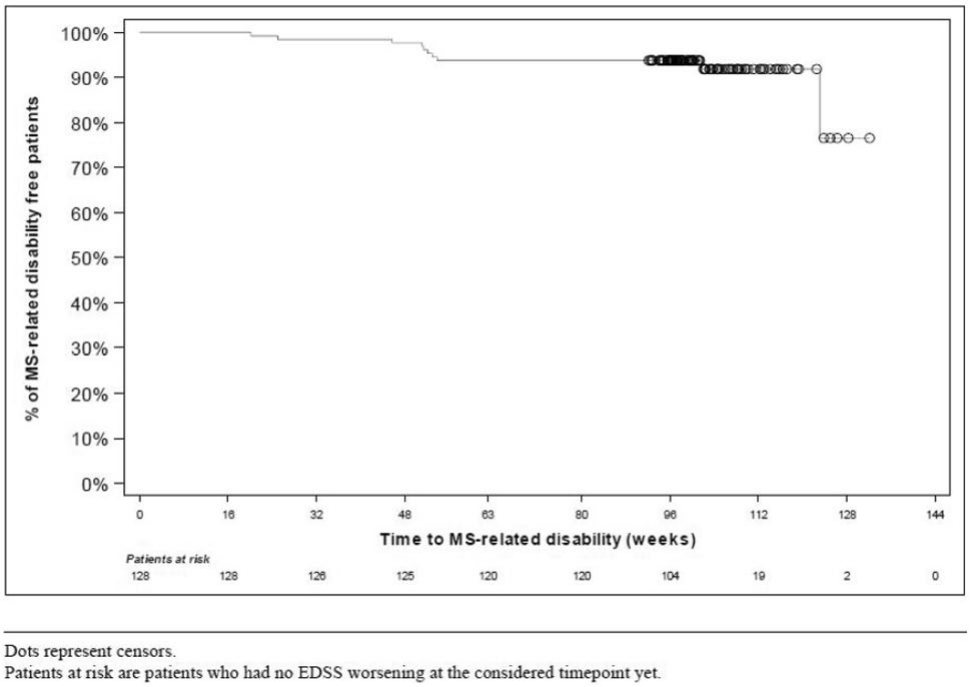
Kaplan–Meier plot of time to MS-related disability

In the FAS, 98.4% of patients (95% CI 94.5; 99.8) were free of disability progression at Week 48 vs. baseline, 94.5% (95% CI 89.1; 97.8) at Week 96 vs. baseline, and 99.2% (95% CI 95.7; 100) at Week 96 vs. Week 48. Fewer patients with a baseline SDMT score ˂35 were free of disability progression than those with SDMT score ≥ 35 when comparing Week 96 to baseline (86.2% vs 96.8%; p = 0.0298) and Week 96 to Week 48 (96.5% vs 100%; p = 0.0692). No remarkable differences were noted when stratifying by gender or naïve status. Mean changes from baseline of EDSS were very close to zero at each visit (− 0.03 ± 0.43 at Visit 2, 0.05 ± 0.49 at Visit 3, 0.05 ± 0.55 at Visit 4, and 0.08 ± 0.54 at Visit 5). Results stratified by sex, naïve/non-naïve status, the combination of the two, and SDMT were similar (data not shown). Roughly 94% of patients had an EDSS score ≤ 4 at all visits (94.5% at Visit 2; 93.8% at Visits 3, 4 and 5). A Kaplan–Meier plot of time to MS-related disability is shown in Fig. 2.

### Imaging

In the FAS, new MRI activity was absent in 88.3% of patients (95% CI 81.4; 93.3) at Week 48 vs. baseline, 67.2% (95% CI 58.3; 75.2) at Week 96 vs. baseline, and 73.4% (95% CI 64.9; 80.9) at Week 96 vs. Week 48. More naïve than non-naïve patients were free from MRI activity at Week 48 vs. baseline (94.1% vs. 84.4%; p = 0.0948), Week 96 vs. baseline (78.4% vs. 59.7%; p = 0.0275) and Week 96 vs. Week 48 (82.4% vs. 67.5%; p = 0.0631). No noteworthy differences were noted stratifying by gender or SDMT at baseline (data not shown).

### Cognition

**Fig. 3:**
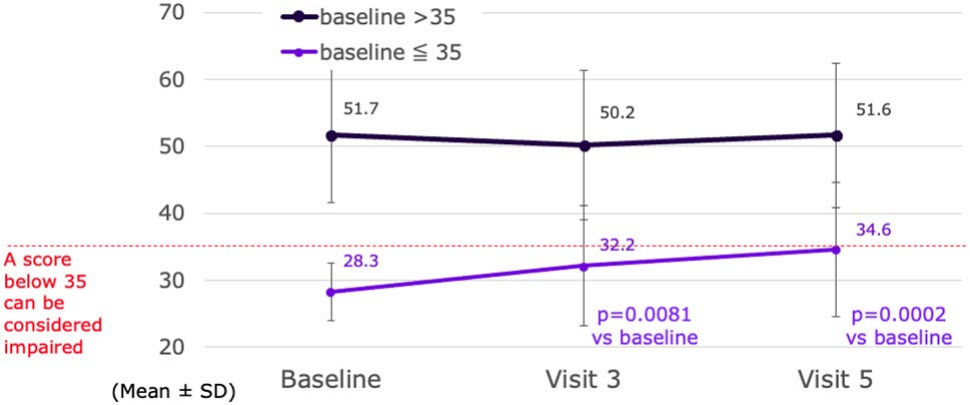
SDMT T-score over time stratified by SDMT score at baseline (FAS population)

In the FAS, SDMT T-score in the overall cohort was stable throughout the study: 46.2 ± 13.4 at baseline, 46.0 ± 13.1 at Week 48 and 47.6 ± 12.9 at Week 96. Stratifying by baseline SDMT T-score shows a stable trend in patients with baseline SDMT ˃ 35 (77% of FAS) and significant improvements in patients with baseline score ≤ 35 (23% of FAS) at Weeks 48 and 96 (Fig. 3). Stable SDMT raw scores were reported for 42.2% and 39.8% of the FAS respectively at Week 48 and Week 96, a similar proportion of patients improved (29.7%) or worsened (28.1%) at Week 48 while more patients improved than worsened at Week 96 (35.9% vs 21.9%).

### NEDA scores

**Fig. 4:**
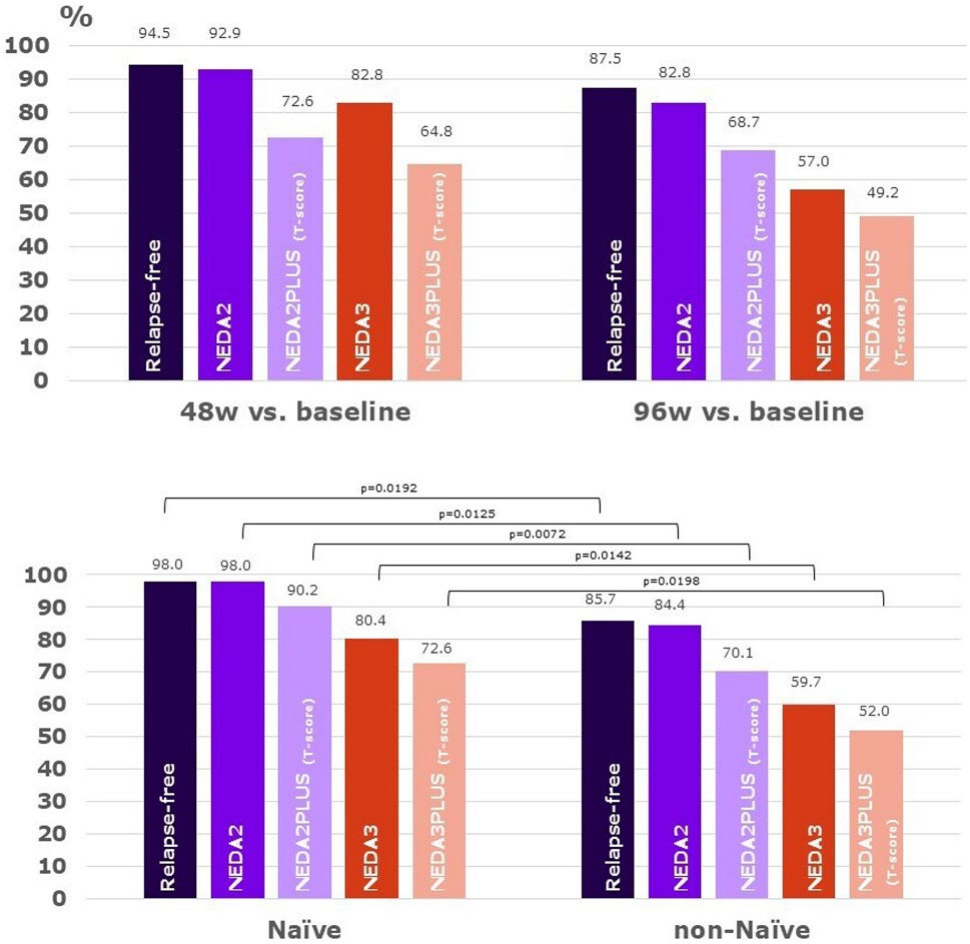
NEDA scores (above the complete FAS population, at the bottom stratified by naïve status at 96 weeks)

The percentages of FAS patients with NEDA are shown in Fig. 4 and range from 92.9% for NEDA-2 to 64.8% for NEDA-3 + at Week 48, and from 82.8% for NEDA-2 to 49.2% for NEDA-3 + at Week 96.

Stratifying by naïve status shows significantly greater proportions of naïve patients achieving NEDA-2 + (p = 0.0545) and NEDA-3 + (p = 0.0332) at Week 96 vs baseline, and NEDA-2 (p = 0.0125), NEDA-2 + (p = 0.0072), NEDA-3 (p = 0.0142) and NEDA-3 + (p = 0.0198) at Week 96 vs Week 48.

The predictiveness of NEDA-3 and NEDA-3 + (raw score) at Week 48 on the change in motor disability at Week 96 did not differ as in both cases outcome was correctly predicted for all patients except one (Supplementary Table 3). All patients except one (99.2%) were free of disability progression at Week 96.

### Persistency, fatigue and depression

**Fig. 5:**
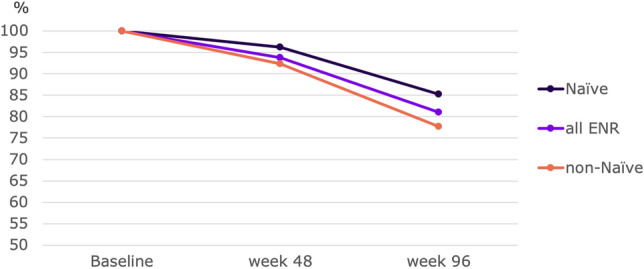
Treatment persistence (ENR population)

In the ENR, 93.8% of patients (95% CI 89.7; 96.7) were treatment persistent at Week 48 and 81.0% (95% CI 75.0; 86.0) were treatment persistent at Week 96. As shown in Fig. 5, greater proportions of naïve patients were treatment persistent at Week 48 (96.3% vs 92.3%) and at Week 96 (86.3% vs 77.7%).

Mean FSS scores were 4.0 ± 1.7 at baseline, 4.0 ± 1.8 at Week 48 and 4.0 ± 1.7 at Week 96. Mean change from baseline was very close to zero at each visit, suggesting no overall progression of fatigue. The proportion of fatigue-free patients (FSS total score > 4.6) was 60.9% at baseline, 60.2% at Week 48 and 58.6% at Week 96.

The proportion of patients free of depression (BDI score < 10) was 75.0% at baseline, 80.5% at Week 48 and 76.6% at Week 96.

### Adverse events

In the SAF, 75 patients (35.7%) experienced at least 1 adverse event (AE) (128 events); 22 (10.5%) at least 1 treatment-related AE (29 events) and 7 (3.3%) at least one serious adverse event (SAE) (7 events). No treatment-related SAEs were reported. Adverse events of special interest (AESI) were reported for two patients (1.0%): one (0.5%) became pregnant and one (0.5%) had hypertension (not treatment related and considered of special interest because it exceeded the threshold values set by the study protocol: systolic blood pressure ≥ 160 mmHg, diastolic blood pressure ≥ 100 mmHg). Twenty-nine AEs were of moderate severity (23 patients, 11.0%) and 2 were severe (arthralgia and gastrointestinal hemorrhage – both SAEs) (2 patients, 1.0%). Eleven patients (5.2%) had treatment-related AEs leading to treatment withdrawal: alopecia and drug ineffectiveness (two patients each), leukopenia, lymphopenia, diarrhea, dyspepsia, peripheral edema, pneumonia, GGT increase and abnormal head MRI (one patient each). The most common treatment-related AEs were hypertension, alopecia and lymphopenia.

## Discussion

This Italian prospective observational study investigated the real-world efficacy of teriflunomide over a 96-week period. Teriflunomide confirmed its efficacy profile in a real world setting as the majority of patients in the overall cohort were free of relapse, new MRI activity, SDP, deteriorating cognitive performance, fatigue or symptoms of depression in the comparisons between 48 weeks and baseline, 96 weeks and baseline, and 96 weeks and 48 weeks. Moreover, EDSS, SDMT, FSS and BDI total scores, as well as mean changes from baseline suggest little or no disease progression during the study. The ARR was extremely low, indicating that patients were practically free from relapse, including patients with disease activity before starting treatment with teriflunomide. Significant proportions of patients achieved NEDA-2 and NEDA-3 + at Week 48 and Week 96, with greater percentages reported for naïve patients. These results confirm those of other observational studies conducted in an Italian population. In the TER-Italy study, the annual relapse rate (ARR) was reduced from 0.56 (0.69) in the pre-teriflunomide year to 0.22 (0.52) on-treatment (− 61%, p < 0.001). Approximately 28% of patients experienced disease activity over a median follow-up of 2.75 years: ~ 9% had relapses but not disability worsening; ~ 13% had isolated disability worsening; and ~ 6% had both relapses and disability worsening [26]. In a study by Lorefice et al., NEDA-3 status was achieved in 58.8% patients at 24 months, and in 56.8% patients at 36 months. No clinical and MRI activity were reported in 175 (85.2%) and 166 patients (81.4%), respectively, while no disability progression was reported in 162 (79.4%) patients and 70.8% of patients were still on treatment after a median follow-up of 2.75 years [27]. Zanghì et al. found no differences between teriflunomide and dimethyl fumarate for all the investigated outcomes: time to first relapse, time to confirmed disability progression, time to discontinuation, and ARR [28].

These encouraging results may be partly related to the presence in our cohort of patients affected by mild forms of the disease and to a high proportion of naïve patients (38%). When compared to previously treated patients, naïve patients were significantly older, had shorter times from onset of symptoms and last relapse, and presented greater disease activity in terms of relapses over the previous two years. Naïve patients had significantly better outcomes in terms of relapses, MRI activity and achievement of NEDA. Naïve patients were also more treatment persistent. In previously treated patients, the switch to teriflunomide was driven mostly by tolerability issues. As in other Italian studies [26, 27], our study suggests that therapy with teriflunomide might be particularly indicated for naïve patients with mild disability or for those who switched initial treatment due to poor tolerability.

NEDA is increasingly recognized as an important treatment goal for patients and achieving NEDA-3 status in the first 2 years of treatment was shown to hold 80–90% positive predictive value for the absence of longer-term disability accrual in the following years [29]. Nevertheless, whether NEDA-3 actually represents the most reliable surrogate marker of disease activity-free status is still under debate [30, 31]. There are several factors which influence treatment outcomes in MS, including the heterogeneous nature of the disease, prognostic factors at disease onset and different individual response to therapies. In RRMS, the concept of NEDA-3 was proposed, but over time this three-parameter endpoint has been challenged. The need to include cognitive impairment and quality of life measures was noted. Our study seems to suggest that adding cognitive performance to the NEDA concept does not increase its ability to predict disability progression in the medium term. However, this is likely due to the fact that all patients except one were free of disability progression at Week 96 It may also be due to an insufficient duration of follow-up and to loss to follow-up and limited FAS population caused by the COVID pandemic.

Regardless of its predictive value, cognitive decline is now widely recognized as a core symptom of MS. Moreover, accumulating evidence reports that less explored cognitive domains (i.e., theory of mind, pragmatics, meta-cognition, prospective memory) might also be affected in the absence of overall CI [32]. Among the cognitive tests used in MS, the SDMT is the most sensitive, likely because good performance depends on multiple functions affected by MS (mostly processing speed, but also memory and visual scanning) [33]. In our study, mean SDMT T-score remained stable in the overall cohort and improved significantly in patients with baseline score ≤ 35. Such improvement (of at least four points) is clinically significant and trended upward over time reaching almost normal values. Furthermore, a stable trend in patients with baseline SDMT ˃ 35 suggests there was no learning effect, although a learning effect only in the population with baseline SDMT score ≤ 35 cannot be excluded. Indeed, the learning effect can be higher in patient with a lower baseline and there is a ceiling effect on learning effect. Nevertheless, this result should be considered exploratory and needs to be confirmed by other studies.

Treatment persistence was high, also considering that patients had been on teriflunomide for at least 24 weeks prior to study entry and is consistent with the efficacy and safety profile of teriflunomide and its patient-friendly use.

Finally, our study confirmed the favourable safety profile of teriflunomide as no treatment-related SAEs were reported and only eleven patients (5.24%) discontinued treatment due to well-known treatment-related AEs.

The limitations of this study are related to substantial loss to follow-up (and reduced FAS population) and missing data, which may be due to the relatively long observation period and the COVID-19 pandemic, and which may have prevented a sound evaluation of the primary endpoint. On the other hand, this also resulted in a relatively high number of patients not entering in the FAS, leading to a potential overestimation of the efficacy of teriflunomide.

## Conclusion

Teriflunomide was found to be safe and effective in a population made up of patients with mild forms of the disease who are naïve to treatment or who have switched from another DMT due to safety concerns. The real-world efficacy of teriflunomide was confirmed in terms of relapses, disability progression and NEDA, with better outcomes for previously untreated patients. Furthermore, cognitive performance remained stable and even improved in patients with lower performance at baseline, although the latter needs to be confirmed by further studies. The SDMT confirmed to be suitable for office visits and should be adopted as routine clinical practice. Treatment persistence to teriflunomide was quite high and may be correlated with its safety and efficacy profile.

The ability of NEDA-3 and NEDA-3 + to predict motor disability, could not be evaluated properly due to the population and/or the duration of follow-up. Further studies aimed at understanding the associations between NEDA and cognitive performance are warranted to better determine the composite measure with the best predictive value.

### Supplementary Information

Below is the link to the electronic supplementary material.

Supplementary file1 (DOCX 28 KB)
