# Supplementary material for: Effectiveness of teriflunomide on No Evidence of Disease Activity and cognition in relapsing remitting multiple sclerosis: results of the NEDA3PLUS study
Source: J Neurol. 2023 Jul 5;270(10):4687–96. doi: 10.1007/s00415-023-11820-0 (PMC10511573; doi:10.1007/s00415-023-11820-0)
Supplement: Supplementary file 1 — Supplementary file1 (DOCX 28 KB) [file 415_2023_11820_MOESM1_ESM.docx]

Supplementary Table 1: reasons for treatment discontinuation according to naïve status

| **Reason for treatment discontinuation** | **Non-naïve (n=130)** | | **Naïve (n=80)** | |
| --- | --- | --- | --- | --- |
|  | **Discontinued (n=32; 24.6%)** | | **Discontinued (n=13; 16.3%)** | |
|  | **No.** | **% of discontinued** | **No.** | **% of discontinued** |
| Adverse Event | 5 | 15.6% | 1 | 7.7% |
| Lack of Efficacy: disability | 1 | 3.1% | 0 | - |
| Lack of Efficacy: MRI | 8 | 25.0% | 6 | 46.2% |
| Lack of Efficacy: other | 1 | 3.1% | 1 | 7.7% |
| Lack of Efficacy: relapse | 5 | 15.6% | 1 | 7.7% |
| Lost to Follow-up | 8 | 25.0% | 3 | 23.1% |
| Medical Decision | 1 | 3.1% | 0 | - |
| Other | 1 | 3.1% | 0 | - |
| Subject Decision | 2 | 6.3% | 0 | - |
| Unexpected Pregnancy | 0 | - | 1 | 7.7% |

Supplementary Table 2: Baseline disease characteristics of Enrolled population and Full Analysis Set

| **Baseline characteristics** | **Enrolled Population (N=210)** | **Full Analysis Set (N=128)** |
| --- | --- | --- |
| Age (years) |  |  |
| Mean (SD) | 47.0 (8.6) | 47.1 (8.7) |
| Gender |  |  |
| Female | 71.4 % | 73.4 % |
| Education (years) |  |  |
| Mean (SD) | 12.5 (3.6) | 12.6 (3.5) |
| EDSS score, mead (SD) | 1.97 (1.33) | 1.85 (1.29) |
| Time from onset of MS symptoms (months) |  |  |
| Mean (SD) | 141.3 (118.3) | 141.5 (121.2) |
| Time from MS diagnosis (months) |  |  |
| Mean (SD) | 100.7 (101.0) | 98.6 (99.3) |
| Number of relapses in the previous year, n (%) |  |  |
| 0 | 124 (59.1) | 75 (58.6) |
| 1 | 72 (34.3) | 44 (34.4) |
| Number of relapses in the last two years, n (%) |  |  |
| 0 | 98 (46.7) | 59 (46.1) |
| 1 | 83 (39.5) | 49 (38.3) |
| Time from onset of last relapse (months) |  |  |
| Mean (SD) | 52.2 (59.0) | 51.9 (59.4) |
| Severity of last relapse, n (%) |  |  |
| Mild | 133 (63.3) | 82 (64.1) |
| Moderate | 73 (34.8) | 44 (34.4) |
| Severe | 4 (1.9) | 2 (1.6) |
| Naïve patients |  |  |
| Yes | 38.1 % | 39.8 % |

Supplementary table 3: predictiveness of NEDA-3 and NEDA-3+ (raw score) at Week 48 on the change in motor disability at Week 96

| **Prediction of disability free**  **(96 vs 48 weeks)** | | **NEDA3** | |
| --- | --- | --- | --- |
|  |  | Incorrect prediction | Correct prediction |
| **NEDA3PLUS** | Incorrect prediction | 1/128 | 0/128 |
|  | Correct prediction | 0/128 | 127/128 |
